# Foot deformity and quality of life among independently ambulating children with spina bifida in South Korea

**DOI:** 10.1186/s12887-023-04100-3

**Published:** 2023-06-05

**Authors:** Hyeseon Yun, Eun Kyoung Choi, Hyun Woo Kim, Jeong Sook Ha, Doo Sung Kim, Kun-Bo Park

**Affiliations:** 1 College of Nursing and Brain Korea 21 FOUR Project, Yonsei University, Seoul, Republic of Korea; 2 College of Nursing and Mo-Im Kim Nursing Research Institute, Yonsei University, 50-1 Yonsei-ro Seodaemun-gu, Seoul, 03722 Republic of Korea; 3 Division of Pediatric Orthopaedic Surgery, Severance Children’s Hospital, Yonsei University College of Medicine, 50-1 Yonsei-ro Seodaemun-gu, Seoul, 03722 Republic of Korea; 4 Severance Children’s Hospital, Yonsei University Health System, Seoul, Republic of Korea

**Keywords:** Child, Foot deformities, Orthopedics, Spinal dysraphism, Quality of life

## Abstract

**Background:**

Children with spina bifida (SB) may have congenital or acquired foot deformities due to neurological defects in the spinal cord. As the musculoskeletal system keeps growing, foot deformities can develop or become aggravated. Thus, healthcare providers should provide constant monitoring and proper orthopedic management. Since foot deformities can affect not only the gait but also the daily life of children with SB, it is necessary to investigate the impact of foot deformities on everyday life. The purpose of this study was to examine the relationship between foot deformity and health-related quality of life (HRQoL) among independently ambulating children with SB.

**Methods:**

This cross-sectional study examined the associations between foot deformity and HRQoL using two patient-reported outcome measures (Oxford Ankle Foot Questionnaire, Pediatric Outcomes Data Collection Instrument) in 93 children with SB aged 7–18 years between January 2020 and July 2021.

**Results:**

Children with foot deformity (n = 54) reported lower scores in all subscales (physical, school and play, emotional, and footwear) of the Oxford Ankle Foot Questionnaire for children than those without foot deformity (n = 39; *p* < 0.001). Additionally, in terms of the Pediatric Outcomes Data Collection Instrument, children with foot deformity also reported poorer scores in four subscales (transfer and basic mobility, sports and physical functioning, comfort and pain, happiness with physical functioning; *p* < 0.001) than those without foot deformity, whereas upper extremity functioning was not significantly affected. Children with foot deformities, particularly those with bilateral foot deformities, equinus deformities, or mixed deformities, which are different types of right and left foot deformities, have a lower perceived HRQoL (*p* < 0.05).

**Conclusions:**

Among independently ambulating children with SB, those with foot deformities showed lower HRQoL. Moreover, children with foot deformities tend to have other clinical problems, including bladder and bowel dysfunction. Therefore, orthopedic management should consider the multifaceted factors that affect children’s daily life and HRQoL.

## Background

Spina bifida (SB) is a congenital anomaly of the central nervous system and is caused by a neural tube defect during early fetal development [1]. Individuals with SB display a wide range of clinical features depending on the level of the neurological lesion, such as neurogenic bladder and/or bowel, hydrocephalus, cognitive impairment, sensory and motor dysfunction in the lower extremities, and orthopedic deformities, including foot deformity, scoliosis, and hip dislocation [1]. Hence, a multidisciplinary approach, which includes neurosurgery, urology, and orthopedic surgery, is essential for the overall management of these children [1]. Regular check-ups are required in children with SB as they grow because they are at risk of recurrence of spinal cord adhesions [2] and neurological complications, such as tethered spinal cord syndrome [3]. Given that spinal nerve damage is irreversible, efforts to maintain an optimal level of neurological function are required for the rest of the life of individuals with SB [4], and they need comprehensive long-term follow-up for chronic conditions.

The clinical characteristics of SB in the Korean population tend to be at the low-lumbar and sacral levels [5], and many individuals with SB which clinically mild severity wherein independently walking is possible without a wheelchair or crutches [6]. Reportedly, only 2.3% of Korean individuals with SB use a wheelchair [6]. However, it has also been reported that 33.7% of young Korean adults with SB have foot deformities, and 23.2% have lower limb weakness [5]. People with SB lesions at the low-lumbar or sacral levels generally develop calcaneus deformities [2]. Pes calcaneus can often cause skin breakdown resulting from weight bearing only on the calcaneus, without weight bearing on the forefoot [7, 8]. Foot deformities also affect gait, and prolonged gait imbalance can cause hip dislocation, rotation of the knee joint, and scoliosis [2, 4]. Furthermore, foot deformity can be congenital or develop over time due to muscle imbalance during growth [2, 8]. Therefore, regular orthopedic follow-up and appropriate management, including orthosis or corrective surgery, are necessary for children with SB.

Orthopedic outcome assessment has mainly been performed using anatomical and functional evaluation methods, such as physical exam, radiography, pedobarography, and computerized gait analysis [2]. In particular, radiography is used to evaluate changes in children’ orthopedic outcomes, and there are many reports of radiological improvement in outcomes after orthopedic management [9, 10]. When evaluating changes in orthopedic conditions or actual gait improvements, pedobarography and gait analysis can be useful to complement radiography, which only shows correction from a static position [8]. Although these objective diagnostic tests can adequately explain anatomical and functional changes, they do not reflect how orthopedic deformities and gait affect daily life and how the effects of orthopedic management influence health-related quality of life (HRQoL) from the perspective of children with SB [11, 12]. Recently, it has been recommended to measure the effects of therapeutic interventions with both objective physical and functional outcomes and subjective HRQoL indicators [12, 13].

However, studies investigating HRQoL in children with SB tended to focus on bladder and/or bowel dysfunction including incontinence [6, 14, 15], and there is limited information on HRQoL related to orthopedic problems. It can be surprising to the attending orthopedist that musculoskeletal problems or deformities that seem quite impressive are far down the list of the patient’s priorities. Furthermore, it has been suggested that further studies address the impact of orthopedic problems and mobility on HRQoL [2]. Therefore, in this study, we aimed to examine the relationship between foot deformity and HRQoL in children with SB.

## Methods

### Aim

This study aimed to examine the relationship between foot deformity and health-related quality of life (HRQoL) among independently ambulating children with SB.

### Study design and participants

This cross-sectional study included children with SB who presented for regular follow-up at the outpatient clinic in the pediatric orthopedic surgery department of the Severance Children’s Hospital from March 2020 to July 2021. Children were enrolled if they were aged 7–18 years. The following exclusion criteria were applied: inability to walk independently (n = 2) and less than two years since corrective surgery (n = 6). The orthopedic surgeon briefly discussed the study with each child and their parents. A research assistant subsequently provided more detailed information for children and parents interested in the study. The survey questionnaire included questions addressing clinical characteristics, the impact of the ankle and foot on children’s daily life and HRQoL.

The sample size required for this study was calculated using G*Power 3.1.9.7; one-way ANOVA; two-sided; effect size = 0.25 to 0.40 (medium to large); α = 0.05; power (1-β) = 0.80; four groups. The sample size was calculated to range from 76 to 180. Since there was no previous study analyzing the differences in HRQoL according to foot deformity in children with SB, we reviewed previous studies that analyzed HRQoL differences in children with SB according to bowel management methods, SB lesion, or incontinence; the number of sample size of each study was 159, 173, and 298 children, respectively [6, 15, 16]. Although the sample size in this study was smaller than that of previous studies, we did our best and aimed to recruit all children who visited the hospital during the study period and met the eligibility criteria. Finally, 93 children were involved in the study. The study was approved by Yonsei University Health System, Severance Hospital, Institutional Review Board (No. 4-2019-1248). All methods were carried out in accordance with the Declaration of Helsinki. Written informed consent was obtained from all children and their parents.

### Measures

#### Impact of foot and ankle conditions

The impact of foot and ankle conditions was measured using the Oxford Ankle Foot Questionnaire (OxAFQ) [17]. OxAFQ has been developed and validated to measure the impact of foot and ankle conditions on aspects of life that are considered important for children [17, 18]. The questionnaire consists of 15 items rated on a 5-point Likert scale (0, always; 1, very often; 2, sometimes; 3, rarely; and 4, never), the first 14 of which are used to calculate subscale scores, and comprises three subscales (physical, 6 items; school and play, 4 items; emotional, 4 items; and footwear as an additional item). The scores for the three subscales are reported separately; therefore, there is no total score. Subscale scores were calculated by deriving the sum of each subscale and subsequently dividing it by the subscale’s maximum value; these scores were subsequently transformed to a percentage (0–100) scale to aid interpretation. A higher score corresponds to better function. The final item, namely, item 15 (“Has your foot or ankle stopped you from wearing any shoes you wanted to wear?”), was added to reflect the concern of many children that they cannot wear the footwear they like. Although this issue is important to children, it psychometrically does not fit into any of the subscales; this final item has been reported separately [17]. Cronbach’s alpha was 0.96 in this part of the study.

#### Health-related quality of life

The Pediatric Outcomes Data Collection Instrument (PODCI) is used to measure the overall musculoskeletal function and HRQoL in children aged 2–18 years with orthopedic conditions, focusing especially on those that are moderate to severe [19]. The Korean version of the PODCI [20] was used to measure HRQoL. The PODCI subscales comprise the following: [1] upper extremity functioning, [2] transfer and basic mobility, [3] sports and physical functioning, [4] comfort and pain, [5] happiness with physical functioning, and [6] global functioning [17]. Global functioning was calculated as the average of the scores of the four subscales except for happiness. The scoring system used for the questionnaire was established using an algorithm designed specifically for use by the research team. Each subscale score was calculated individually and standardized (range, 0–100). A higher standardized score indicated better condition. Cronbach’s alpha was 0.907 in this study.

### Data collection

The research assistant screened the list of children with SB who met the eligibility criteria and contacted them and/or their parents by telephone to explain the purpose of the study and its procedures. Children who were willing to participate in this study were provided with a copy of the survey by the research assistant when they visited the clinic for follow-up. Before they completed the survey, the researcher explained it to them in detail. We obtained the OxAFQ and PODCI questionnaires; additionally, demographic and clinical information, including sex, age, diagnosis, neurological lesion, functional classification, type of orthopedic deformity (hip, spine, knee, and foot), presence of a ventriculoperitoneal (V-P) shunt, type and date of orthopedic surgery, voiding and defecation methods, and urinary/fecal incontinence, were obtained from the electronic medical records by the research assistants.

A pediatric orthopedic surgeon with 21 years of experience described the spine, hip, knee, or foot deformities in the electronic medical record during the physical examination. A pediatric orthopedic surgeon with at least 15 years of experience categorized the eight types of foot deformity (equinocavovarus, cavovarus, calcaneus, planovalgus, calcaneocavovarus, calcaneocavus, equinus, and vertical talus) and classified them into four types of foot deformity (calcaneus, equinus, cavus, and planus) to analyze the differences in HRQoL according to foot deformity types.

### Statistical analyses

Statistical analyses were performed using SPSS version 26.0 (IBM Corp., Armonk, NY, USA), and values of *p* < 0.05 were considered statistically significant in this study. The clinical characteristics of the study population were examined according to foot deformity status using descriptive statistics and Pearson’s chi-squared test. Additionally, the mean difference in OxAFQ and PODCI scores was analyzed using an independent t-test, one-way ANOVA, Kruskal–Wallis one-way analysis of variance based on the clinical characteristics, including foot deformity status.

## Results

### Clinical characteristics of study participants

A total of 93 children with SB were included in this study. The clinical characteristics of the study participants were investigated according to foot deformity status; 58.0% of the children had a prominent foot deformity, and 42.0% had no clinically significant foot deformity (Table 1). A total of 62 participants were boys (66.7%); 54 children (58.0%) were aged 7-12 years, and 39 children (42.0%) aged 13-18 years. The average age was 11.9 ± 3.6 years. The population of children diagnosed with lipomeningomyelocele (79.6%) was significantly higher than that of those diagnosed with myelomeningocele (20.4%). There were no significant differences in gender, age, and diagnosis distribution between children with deformed feet and those with normal feet. According to SB classification [21], 16 children (17.2%) had low-lumbar (L4-L5), 47 (50.5%) had high-sacral (S1-S2), and 30 (32.2%) had low-sacral (S3-S5) SB lesions. There was a significant difference in the distribution of SB lesions spinal level according to the presence or absence of foot deformity (*χ*^2^ = 32.002, *p* < 0.001). Children with normal feet had more SB lesions in the low-sacral (26.9%) level, followed by high-sacral (12.9%) and low-lumbar (2.1%) levels and children with foot deformities had more SB lesions in the high-sacral (37.6%) level, followed by low-lumbar (15.1%) and low-sacral (5.4%) levels. In addition, children with foot deformity had significantly higher rates of V-P shunt (6.5%), clean intermittent catheterization (CIC) (38.7%), and enema (22.6%) when compared to children with normal foot; only 8.6% and 5.4% of children with normal feet urinated using CIC (*χ*^2^ = 4.632, *p* = 0.031) and defecated through enema (*χ*^2^ = 19.351, *p* < 0.001), respectively, and none had V-P shunt (*χ*^2^ = 7.64, *p* = 0.006). There were 38 (40.9%) and 18 (19.4%) children with urinary and fecal incontinence, respectively, with no differences according to the presence of foot deformity.

**Table 1:** Clinical characteristics of study participants according to presence or absence of foot deformity

Variable	Category	Total	Normal foot	Foot deformity	*χ* ^*2*^ *(p*-value)
		n (%)	n (%)	n (%)	
Total		93 (100.0)	39 (42.0)	54 (58.0)	
Gender	Boy	62 (66.7)	24 (25.8)	38 (40.9)	0.795 (0.373)
	Girl	31 (33.3)	15 (16.1)	16 (17.2)	
Age (years)	Mean (SD)	11.9 (3.6)			
	7–12	54 (58.0)	27 (29.0)	27 (29.0)	3.439 (0.064)
	13–18	39 (42.0)	12 (13.0)	27 (29.0)	
Diagnosis	MMC	19 (20.4)	5 (5.4)	14 (15.0)	2.393 (0.122)
	LMMC	74 (79.6)	34 (36.6)	40 (43.0)	
SB classification	Low-lumbar	16 (17.2)	2 (2.1)	14 (15.1)	**32.002** **(< 0.001)**
	High-sacral	47 (50.5)	12 (12.9)	35 (37.6)	
	Low-sacral	30 (32.3)	25 (26.9)	5 (5.4)	
V-P shunt	No	87 (93.5)	39 (41.9)	48 (51.6)	**4.632** **(0.031)**
	Yes	6 (6.5)	0 (0.0)	6 (6.5)	
Urination method^†^	Spontaneous	52 (55.9)	31 (33.3)	21 (22.6)	**15.142** **(< 0.001)**
	CIC	44 (47.3)	8 (8.6)	36 (38.7)	**19.351** **(< 0.001)**
Defecation method^†^	Spontaneous	70 (75.3)	34 (36.6)	36 (38.7)	**5.119** **(0.024)**
	Enema	26 (28.0)	5 (5.4)	21 (22.6)	**7.64** **(0.006)**
	Stoma	1 (1.1)	0 (0.0)	1 (1.1)	0.055 (0.815)
Urinary incontinence	No	55 (59.1)	27 (29.0)	28 (30.1)	2.83 (0.092)
	Yes	38 (40.9)	12 (12.9)	26 (28.0)	
Fecal incontinence	No	75 (80.6)	35 (37.6)	40 (43.0)	3.562 (0.059)
	Yes	18 (19.4)	4 (4.3)	14 (15.1)	

^†^Multiple response items. Values that are statistically significant at *p* < 0.05 are emboldened. CIC, clean intermittent catheterization; LMMC, Lipomyelomeningocele; MMC, Myelomeningocele; SB, spina bifida; SD, standard deviation; V-P shunt, ventriculo-peritoneal shunt

### Orthopedic characteristics of study participants

Table 2 presents the orthopedic characteristics of the study participants. Among the 93 children with SB, 58.0% of the children had a prominent foot deformity, and 42.0% had normal foot. Of the 54 children with foot deformity, 23 children (24.7%) underwent corrective surgery, and 31 children (33.3%) did not undergo corrective surgery. The number of children with both bilateral and unilateral foot deformities was 27 (29%). Eight subtypes of foot deformities were classified into four types: calcaneus, equinus, cavus, and planus. Equinus or calcaneus deformities have a stronger effect on gait because these are related to push-off at the terminal stance phase and foot clearance during the swing phase. The most common type of foot deformity was calcaneus (17.2%), followed by equinus (15%), cavus (15%), and planus (6.5%). In addition, four children had mixed deformities, which are different pairs of right and left foot deformities, including calcaneocavus-planovalgus, planovalgus-calcaneocavovarus, planovalgus-equinocavovarus, and equinocavovarus-planovalgus.

**Table 2:** Orthopedic characteristics of study participants (*N* = 93)

Variable	Category		n (%)
Type of foot	Normal foot		39 (42.0)
	Foot deformity		54 (58.0)
Foot corrective surgery^†^ (n = 54)	No		31 (33.3)
	Yes		23 (24.7)
Location of foot deformity^†^ (n = 54)	Bilateral feet		27 (29.0)
	Unilateral foot		27 (29.0)
		Right foot	13 (14.0)
		Left foot	14 (15.0)
Type of foot deformity^†^ (n = 54)	(1) Calcaneus		16 (17.2)
		Calcaneus	9 (9.7)
		Calcaneocavus	2 (2.1)
		Calcaneocavovarus	5 (5.4)
	(2) Equinus		14 (15.0)
		Equinus	2 (2.1)
		Equinocavovarus	12 (12.9)
	(3) Cavus		14 (15.0)
		Cavovarus	14 (15.0)
	(4) Planus		6 (6.5)
		Planovalgus	4 (4.3)
		Vertical talus	2 (2.2)
	(5) Mixed		4 (4.3)
Wearing orthosis	No		49 (52.7)
	Yes		44 (47.3)
Type of orthosis^†^ (n = 44)	AFO		40 (43.0)
	Metatarsal pad		2 (2.2)
	Arch support		2 (2.2)
Hip deformity	Normal		91 (97.8)
	Dislocation		2 (2.2)
Knee deformity	Normal		91 (97.8)
	Flexion contracture		2 (2.2)
Scoliosis	< 40°		91 (97.8)
	≥ 40°		2 (2.2)

^†^Subgroup analysis. AFO, Ankle-foot orthosis

Among the 93 children in this study, 44 (47.3%) children who wore orthosis. Specifically, 11 children with low-lumbar lesions, 27 children with high-sacral lesions, and 2 children with low-sacral lesions needed ankle-foot orthoses (n = 40, 43%). Two children with low-lumbar lesions used metatarsal pads (2.2%) due to plantar callosity, and two children with high-sacral lesions used arch support (2.2%) due to planovalgus. Most participants did not have other clinically significant bony deformities in the hip, knee, or spine. Hip dislocation, knee flexion contracture, and scoliosis greater than 40 degrees were found each in two children (2.2%).

### Differences in mean OxAFQ-C scores by foot type

We examined the differences in mean scores of OxAFQ-C according to the type of foot deformity (Table 3). Children with normal feet (n = 39) had significantly higher scores on all OxAFQ-C subscales than those with foot deformity (n = 54) (physical, *t* = 6.623, *p* < 0.001; school and play, *t* = 4.819, *p* < 0.001; and emotional, *t* = 6.805, *p* < 0.001; *t* = 7.631, *p* < 0.001). Except for footwear, all OxAFQ-C subscale scores were significantly higher in children with unilateral foot deformity than in those with bilateral foot deformity (physical, *t* = 2.139, *p* = 0.038; school and play, *t* = 3.059, *p* = 0.004; emotional, *t* = 2.439, *p* = 0.018). However, children with SB who underwent foot corrective surgery had lower OxAFQ-C scores than those without foot corrective surgery in all subscales, but the differences were not statistically significant. In addition, the differences in OxAFQ-C scores in all subscales according to foot deformity types were not significant.

**Table 3:** Differences in mean scores of the OXAFQ-C by foot type (*N* = 93)

Variable	Category	n	Subscale of the OxAFQ-C							
			Physical		School and Play		Emotional		Footwear	
			M (SD)	*t/F* (*p*-value)	M (SD)	*t/F* (*p*-value)	M (SD)	*t/F* (*p*-value)	M (SD)	*t/F* (*p*-value)
Type of foot	Normal foot	39	87.9 (16.1)	**6.623** **(< 0.001)**	94.2 (12.7)	**4.819** **(< 0.001)**	95.0 (9.9)	**6.805** **(< 0.001)**	91.0 (18.6)	**7.631** **(< 0.001)**
	Foot deformity	54	59.3 (25.5)		76.4 (22.7)		67.5 (27.3)		44.9 (38.7)	
Foot corrective surgery^†^ (n = 54)	No	31	62.4 (23.9)	1.014 (0.315)	80.3 (19.5)	1.4 (0.169)	69.4 (25.0)	0.581 (0.564)	48.4 (35.3)	0.765 (0.448)
	Yes	23	55.3 (27.5)		71.2 (26.0)		65.0 (30.6)		40.2 (43.1)	
Location of foot deformity^†^ (n = 54)	Unilateral	27	66.5 (29.3)	**2.139** **(0.038)**	85.2 (19.6)	**3.059** **(0.004)**	76.2 (26.5)	**2.439** **(0.018)**	49.1 (45.2)	0.789 (0.434)
	Bilateral	27	52.2 (19.0)		67.6 (22.5)		58.8 (25.8)		40.7 (31.1)	
Type of foot deformity^†^ (n = 54)	Calcaneus	16	63.3 (18.6)	2.932 (0.569)	78.5 (23.5)	2.213 (0.697)	70.7 (24.8)	3.862 (0.425)	48.4 (33.5)	3.569 (0.467)
	Equinus	14	58.9 (32.3)		75.9 (24.4)		70.1 (30.4)		42.9 (43.2)	
	Cavus	14	61.3 (26.4)		78.6 (25.5)		68.8 (33.1)		55.4 (42.9)	
	Planus	6	59.0 (23.5)		74.0 (15.5)		62.5 (19.0)		33.3 (37.6)	
	Mixed	4	38.6 (24.6)		65.6 (18.8)		48.5 (12.9)		18.8 (23.9)	

^†^Subgroup analysis. Values that are statistically significant at *p* < 0.05 are emboldened

### Differences in mean PODCI scores by foot type

We examined the differences in mean scores of PODCI according to the type of foot (Table 4). The PODCI scores according to the type of foot followed a pattern similar to that of the OxAFQ-C. Except for upper extremity functioning, all PODCI subscale scores were significantly higher in children with normal foot (transfer and basic mobility, *t* = 3.137, *p* = 0.003; sports and physical functioning, *t* = 5.393, *p* < 0.001; comfort and pain, *t* = 2.082, *p* = 0.04; happiness with physical condition, *t* = 4.175, *p* < 0.001). As for the differences in mean PODCI scores according to foot deformity type (n = 54), PODCI score for equinus deformity was significantly lower than that of calcaneus, cavus, and planus deformities in transfer and basic mobility, and in upper extremity functioning (f = 13.724, p < 0.008). For mixed foot deformity type, PODCI scores were significantly lower than those of calcaneus and cavus deformities (f = 13.724, p < 0.014). However, children with SB who underwent foot corrective surgery had lower PODCI scores than those without foot corrective surgery in all subscales, except for happiness with physical condition, but the difference was not statistically significant. In addition, children with bilateral foot deformity had lower PODCI scores in all subscales, except comfort and pain than children with unilateral foot deformity, but the difference was not statistically significant.

**Table 4:** Differences in mean scores of the PODCI by foot type (*N* = 93)

Variable	Category	n	Subscale of the PODCI											
			Upper extremity functioning		Transfer and Basic mobility		Sports and Physical functioning		Comfort and pain		Global functioning		Happiness with physical condition	
			M (SD)	*t/F* (*p*-value)	M (SD)	*t/F* (*p*-value)	M (SD)	*t/F* (*p*-value)	M (SD)	*t/F* (*p*-value)	M (SD)	*t/F* (*p*-value)	M (SD)	*t/F* (*p*-value)
Type of foot	Normal foot	39	97.8 (7.9)	-0.729 (0.468)	99.5 (1.8)	**3.137** **(0.003)**	95.8 (7.8)	**5.393** **(< 0.001)**	92.0 (13.8)	**2.082** **(0.04)**	96.3 (5.9)	**3.875** **(< 0.001)**	91.4 (13.2)	**4.175** **(< 0.001)**
	Foot deformity	54	98.6 (2.9)		97.0 (5.5)		79.6 (20.0)		85.2 (17.8)		90.1 (9.4)		76.8 (20.6)	
Foot corrective surgery^†^ (n = 54)	No	31	99.1 (2.1)	1.211 (0.235)	97.3 (4.8)	0.405 (0.687)	81.9 (18.1)	0.951 (0.346)	85.3 (16.5)	0.045 (0.965)	90.9 (8.2)	0.685 (0.497)	72.7 (22.2)	-1.692 (0.497)
	Yes	23	98.0 (3.7)		96.7 (6.4)		76.6 (22.5)		85.1 (19.8)		89.1 (11.0)		82.2 (17.2)	
Location of foot deformity^†^ (n = 54)	Unilateral	27	98.1 (3.1)	-1.166 (0.249)	97.9 (3.8)	1.211 (0.231)	81.4 (20.3)	0.639 (0.525)	84.5 (21.5)	-0.266 (0.791)	90.5 (10.6)	0.299 (0.766)	79.1 (21.7)	0.823 (0.414)
	Bilateral	27	99.1 (2.7)		96.1 (6.7)		77.9 (20.0)		85.8 (13.7)		89.7 (8.3)		74.4 (19.6)	
Type of foot deformity^†^ (n = 54)	Calcaneus^a^	16	99.0 (2.4)	**13.724** **(0.008)** ^**††**^	98.1 (4.1)	**12.512** **(0.014)** ^†††^	86.2 (14.3)	4.734 (0.316)	89.8 (11.2)	2.765 (0.598)	93.3 (6.7)	4.964 (0.291)	77.8 (21.7)	0.848 (0.932)
	Equinus^b^	14	96.4 (4.3)		96.6 (4.1)		70.6 (24.7)		78.4 (22.4)		85.5 (11.6)		76.8 (19.7)	
	Cavus^c^	14	100.0 (0.0)		98.8 (4.5)		83.7 (20.2)		86.8 (20.6)		92.3 (9.2)		76.4 (24.8)	
	Planus^d^	6	100.0 (0.0)		96.4 (3.6)		78.1 (13.2)		84.5 (14.7)		89.8 (5.5)		71.7 (16.3)	
	Mixed^e^	4	97.9 (2.4)		89.2 (12.5)		73.3 (25.2)		86.0 (16.7)		86.6 (12.0)		81.3 (16.5)	

^†^Subgroup analysis. Values that are statistically significant at *p* < 0.05 are emboldened. Post-hoc test: ^††^b < a, c, d; ^†††^e < a, c

## Discussion

We investigated the orthopedic characteristics of children aged 7–18 years with SB focusing on foot deformities. The prevalence of foot deformity was higher than that of other orthopedic deformities, such as spine, hip, and knee, and children with foot deformities had a high proportion of bladder or bowel dysfunction and SB lesions at high-sacral or low-lumbar levels. Children with bilateral foot deformity, equinus foot deformity type, or mixed deformity types reported lower HRQoL. These findings suggest that, even children with clinically mild severity of sacral-level SB lesions, may have poor HRQoL if they have specific foot deformities combined with other clinical problems. The study highlights the need to pay attention to orthopedic characteristics that have been relatively overlooked when assessing HRQoL in children with SB.

The most common type of the foot deformity was pes calcaneus. This is consistent with previous findings that individuals with sacral-level SB lesions have a high incidence of pes calcaneus deformities [13]. Foot deformity is associated with the level of SB lesions, with a higher incidence of calcaneus observed in children with lower levels of SB lesions [2, 13]. This results from inadequate innervation in the antagonistic muscle groups, such as the soleus and gastrocnemius, which work against the tibial muscles innervated from the lumbar and sacral regions. Furthermore, insufficient innervation of tibial muscles can result in overactivity of the opposing muscles and lead to foot deformities [2, 10]. Pes calcaneal deformity typically results in the breakdown of the skin on the heel and osteomyelitis [2, 8]; therefore, careful monitoring for skin integrity is essential.

Most Korean children with SB can walk independently since their lesions at the sacral level, which are known to not significantly affect ambulation status. However, previous studies of HRQoL in children with SB have identified that factors such as urinary and fecal incontinence requiring CIC or the use of enemas negatively affect HRQoL [6, 15]. In this study, the incidence of bladder or bowel dysfunctions varied according to the presence of foot deformity. Individuals with SB need to be evaluated and managed very carefully because of their multiple comorbidities [2] and an individualized approach is needed to establish an appropriate care plan.

Children with foot deformities tended to have lower mean scores in the footwear subscale of OxAFQ-C, regardless of foot deformity type. Although not statistically significant due to very large standard deviation, these results suggest that children with SB with foot deformities can feel notably limited in choosing the shoes they want. A study on the experience of young adults with SB in Korea found that wearing orthosis and lower limb appearance had a negative effect on body image and psychosocial well-being [22, 23]. This suggests that concerns about appearance and body image related to lower extremity deformities or orthosis use may be as important as mobility problems for children with SB.

Children with foot deformity had a significantly greater mean differences in PODCI scores related to happiness with physical condition compared to physical function when compared to children with normal feet, indicating a potential disparity between perceived physical function and the associated satisfaction. Furthermore, the large standard deviation in the happiness with physical condition scores for children with foot deformities highlights the significance of individual variations in their subjective experiences. As such, assessing the HRQoL of children with SB requires evaluating both physical function and psychosocial well-being [24]. These results align with the disability paradox, where individuals with mild SB tend to compare themselves to healthy peers, resulting in frustration and disappointment [25, 26]. Therefore, high-functioning children with SB in Korea may require attention and support to address their psychosocial vulnerabilities.

This study has some limitations. First, it is necessary to measure improvement in HRQoL according to changes in foot deformity and lower extremity function before and at least 2 years after corrective surgery in the same children; however, we could not evaluate this in our study because of its cross-sectional design. In the future, a longitudinal study with a long-term follow-up is needed. Second, children in the study had a higher percentage of lipomyelomeningocele and were highly functioning, unlike the clinical distribution of SB types in the West [27]. Because prenatal screenings are more frequently performed in South Korea, fetal malformations are more easily detected [6]. Unfortunately, fetuses with myelomeningocele are often terminated, which is not legal but often occurs [6]. Therefore, our findings are difficult to generalize for adolescents with SB with lower functioning. Lastly, neurogenic bladder or bowel is a well-known factor affecting HRQoL and an important confounding factor in this study. To better study the association between foot deformity and HRQoL, it would be appropriate to include only individuals without bladder or bowel dysfunction, but this was not possible in this study because of the small sample size. Therefore, the results of this study should be interpreted with caution, considering that bladder or bowel dysfunction was not adjusted for in the analyses.

## Conclusions

The goal of orthopedic treatment in children with SB is to maintain optimal musculoskeletal status as far as possible for a given individual’s neurological function. In this study, we found that foot deformity is related to poorer HRQoL in children with SB. However, not all children have the same orthopedic needs. Our results suggest that children with foot deformities, particularly those with bilateral foot deformities, equinus deformities, or mixed deformities, have a lower perceived HRQoL. Regarding the HRQoL of children with SB, we identified the need to assess orthopedic characteristics that have not been a concern. Pediatric orthopedists are required to assess the special needs or difficulties of children with SB and take them in consideration when devising the treatment plan. Further studies involving larger populations are needed to explore the differences in HRQoL according to the type of deformity. Therefore, it is not possible to conclude about the need to correct certain deformities, and treatments should be individualized to ensure a plantigrade braceable foot.

## Data Availability

The data that support the findings of this study are available from the corresponding author, upon reasonable request.

## Abbreviations

CIC: Clean intermittent catheterization
HRQoL: Health-related quality of life
LMMC: Lipomyelomeningocele
MMC: Myelomeningocele
OxAFQ-C: Oxford Ankle Foot Questionnaire for Children
PODCI: Pediatric Outcomes Data Collection Instrument
SB: Spina bifida
VP: Ventriculoperitoneal
